# Functional and Molecular Analysis of Proprioceptive Sensory Neuron Excitability in Mice

**DOI:** 10.3389/fnmol.2020.00036

**Published:** 2020-05-05

**Authors:** Jessica F. Madden, Olivia C. Davis, Kieran A. Boyle, Jacqueline A. Iredale, Tyler J. Browne, Robert J. Callister, Douglas W. Smith, Phillip Jobling, David I. Hughes, Brett A. Graham

**Affiliations:** ^1^School of Biomedical Sciences and Pharmacy, Faculty of Health and Medicine, University of Newcastle, Callaghan, NSW, Australia; ^2^Hunter Medical Research Institute (HMRI), New Lambton Heights, NSW, Australia; ^3^Institute of Neuroscience Psychology, College of Medical, Veterinary and Life Sciences, University of Glasgow, Glasgow, United Kingdom

**Keywords:** parvalbumin, proprioception, dorsal root ganglia, sensory neuron, action potential, I_h_, HCN channel

## Abstract

Neurons located in dorsal root ganglia (DRG) are crucial for transmitting peripheral sensations such as proprioception, touch, temperature, and nociception to the spinal cord before propagating these signals to higher brain structures. To date, difficulty in identifying modality-specific DRG neurons has limited our ability to study specific populations in detail. As the calcium-binding protein parvalbumin (PV) is a neurochemical marker for proprioceptive DRG cells we used a transgenic mouse line expressing green fluorescent protein (GFP) in PV positive DRGs, to study the functional and molecular properties of putative proprioceptive neurons. Immunolabeled DRGs showed a 100% overlap between GFP positive (GFP+) and PV positive cells, confirming the PVeGFP mouse accurately labeled PV neurons. Targeted patch-clamp recording from isolated GFP+ and GFP negative (GFP−) neurons showed the passive membrane properties of the two groups were similar, however, their active properties differed markedly. All GFP+ neurons fired a single spike in response to sustained current injection and their action potentials (APs) had faster rise times, lower thresholds and shorter half widths. A hyperpolarization-activated current (I_h_) was observed in all GFP+ neurons but was infrequently noted in the GFP− population (100% vs. 11%). For GFP+ neurons, I_h_ activation rates varied markedly, suggesting differences in the underlying hyperpolarization-activated cyclic nucleotide-gated channel (HCN) subunit expression responsible for the current kinetics. Furthermore, quantitative polymerase chain reaction (qPCR) showed the HCN subunits 2, 1, and 4 mRNA (in that order) was more abundant in GFP+ neurons, while HCN 3 was more highly expressed in GFP− neurons. Likewise, immunolabeling confirmed HCN 1, 2, and 4 protein expression in GFP+ neurons. In summary, certain functional properties of GFP+ and GFP− cells differ markedly, providing evidence for modality-specific signaling between the two groups. However, the GFP+ DRG population demonstrates considerable internal heterogeneity when hyperpolarization-activated cyclic nucleotide-gated channel (HCN channel) properties and subunit expression are considered. We propose this heterogeneity reflects the existence of different peripheral receptors such as tendon organs, muscle spindles or mechanoreceptors in the putative proprioceptive neuron population.

## Introduction

Our experience of somatosensory stimuli such as proprioception, pain, and touch, results from the detection of stimuli in the periphery by specialized receptors before the propagation of these signals along primary afferents. Primary afferents are the peripheral axons of pseudo-unipolar sensory neurons, whose cell bodies are clustered in dorsal root ganglia (DRG). These neurons are responsible for the transmission of sensory information into the dorsal horn (DH) of the spinal cord and on to higher brain structures (Krames, 2014). Importantly, the anatomical positioning of the DRG outside the blood-nerve barriers of the central nervous system (CNS; Hu and McLachlan, 2002), has made sensory neurons a prime target for altering incoming sensory signals, especially those associated with nociception or pain. In particular, pharmacological (anti-inflammatory steroids; Manchikanti, 2000; Vad et al., 2002), neuromodulation *via* electrical stimulation (Deer et al., 2013) and physical interventions such as ganglionectomy (Acar et al., 2008), radio-frequency ablation (Nash, 1986; de Louw et al., 2001) and pulsed-radio frequency activation (Van Zundert et al., 2007) have targeted sensory neurons to treat aberrant sensory signaling.

These above intervention strategies, however, do not account for the fact that sensory neurons are a heterogeneous population and carry different types of information. Most studies on DRG signaling have focused on nociception where nociceptive sensory neurons are distinguished by their small soma diameters, unmyelinated or lightly myelinated axons, and nociceptor-specific molecular markers such as transient receptor potential channels, TRPV1 and TRPA1 (Berta et al., 2017). The same approach has rarely been applied to the other modalities transmitted *via* sensory neurons such as proprioception, which relays information about movement and body position, from peripheral receptors located in muscle, tendon, and joints (Delhaye et al., 2018). Broadly, proprioceptive sensory neurons have been identified by their typically large neuron diameter, axon myelination and fast conduction velocities vs. the smaller sensory neurons that transmit light touch, temperature, and nociception (Lawson, 2005).

The calcium-binding protein parvalbumin (PV) has been used to mark proprioceptive sensory neurons in rodents because it is expressed in large diameter DRG cells (Celio, 1990; Ichikawa et al., 1994, 2004; Honda, 1995; Arber et al., 2000; de Nooij et al., 2013). PV is co-localized with Tyrosine receptor kinase C (Trk C) and Neurotrophin-3 proteins, which are linked to the development of proprioceptive receptors and their primary afferent neurons (Ernfors et al., 1994). Additionally, PV is selectively expressed in muscle spindle afferents in the periphery (Ichikawa et al., 2004). Together, these findings suggest PV is a reliable neurochemical marker for proprioceptive DRG neurons.

The rationale for characterizing the properties of DRG neuron subtypes is to characterize unique properties (e.g., ion channel subtypes) that might be targeted by therapeutic agents to alter sensory function. Similar to whole DRG targeting, this approach has been primarily applied in the pain field. For example, sodium channel blockers, which target specific channel types, are now in clinical trials (Haberberger et al., 2019). Recently, hyperpolarization-activated cyclic nucleotide-gated (HCN) channels and the hyperpolarization-activated current (I_h_) they mediate have been implicated in chronic pain states (Young et al., 2014; Tsantoulas et al., 2017; Lainez et al., 2019). This inward current is activated at hyperpolarized potentials and plays a role in adjusting resting membrane potential and generating rhythmic action potentials (APs; Hughes et al., 2013).

Interestingly, several studies have shown that I_h_ is present in both large (presumably proprioceptive) and small (nociceptive) diameter neuronal populations. Their specific electrophysiological properties, however, are known to differ (Doan and Kunze, 1999; Gao et al., 2012). This suggests sensory neurons of different modalities express a unique pattern of hyperpolarization activated cyclic nucleotide gated (HCN) channels that form tetramers and can be comprised of four distinct subunits, HCN1-4. Until recently, specific blocking agents for HCN subunits have not been described and their varied expression in a variety of tissues has limited our ability to target these channels. More recent studies, however, have reported subunit-specific blockers for HCN 1 and 2 over HCN 4, and these are currently being explored as a pain therapy (Dini et al., 2018). This work underscores the importance of understanding the expression pattern of HCN subunits across modality-specific sensory neuron populations. Importantly, the role of I_h_ in proprioceptive sensory neurons, identified by characteristics other than size, has not yet been investigated.

To address this gap in our knowledge, we used a transgenic mouse line expressing green fluorescent protein (GFP) under a PV promoter gene (PV-eGFP) to study the functional and molecular properties of putative proprioceptive sensory neurons. Targeted-patch clamp recording was undertaken on isolated green fluorescent protein positive (GFP+) and green fluorescent protein negative (GFP−) sensory neurons to study their electrophysiological properties with a focus on I_h_ currents in each population. We also undertook quantitative polymerase chain reaction (qPCR) and immunolabeling analysis to compare the expression of HCN subunits 1–4.

## Experimental Procedures

### Dissection and Preparation of Dissociated Cells

All experiments carried out at the University of Newcastle were in accordance with the Animal Research Act 1985 (NSW), under the guidelines of the National Health and Medical Research Council Code for the Care and Use of Animals for Scientific Purposes in Australia (2013). All experiments carried out in Glasgow were in accordance with the European Community directive 86/609/EEC and UK Animals (Scientific Procedures) Act 1986. We used a BAC transgenic BALB/c mouse (PVeGFP) generated to express an enhanced fluorescent protein (EGFP) under the control of the PV gene promoter (Meyer et al., 2002). The initial description of this mouse showed eGFP was selectively expressed in PV neurons throughout the nervous system. We have also used this mouse previously to characterize PV-positive cells in the DH of the spinal cord (Hughes et al., 2012; Gradwell et al., 2017). Some experiments also used tissue from C57Bl/6 mice for additional immunolabeling analysis.

Adult PVeGFP mice (eight male, nine female, average body weight = 24.4 g) were deeply anesthetized with Ketamine (100 mg/kg i.p, Troy Laboratories, NSW, Australia) and sacrificed *via* rapid decapitation. Lumbar DRGs (L2-L6) from both sides were quickly removed with the aid of a dissecting microscope and placed in substituted artificial cerebrospinal fluid (sACSF) containing in mM: 236 Sucrose, 25 NaHCO_3_, 11 glucose, 1 NaH_2_PO_4_, 2.5 KCl, 2.5 CaCl_2,_ and 1 MgCl. For both electrophysiology and molecular experiments, DRGs were further dissociated into a single cell suspension. Briefly, DRGs were transferred to a HEPES based collagenase solution (10 mg/ml, Worthington Pty Ltd) for 40–60 min at 37°C. Three flame polished glass Pasteur pipettes of decreasing diameter were then used to mechanically triturate the tissue and form a dissociated cell suspension (10× for each pipette diameter). The suspension was washed, and the supernatant replaced with a fresh HEPES solution. In patch-clamp recording experiments the cell suspension (100 μl) was pipetted into a 22 mm plastic Petri dish. Recording commenced after cells had settled and adhered to the bottom of the Petri dish (~15 min), which also acted as the recording bath. For all gene expression analysis, the dissection and dissociation process remained the same, except all solutions, were prepared using diethylpyrocarbonate (DEPC) treated water (0.1%, Sigma Aldrich Pty Ltd), and underwent 24 h of agitation followed by autoclaving (120°C for 30 min) to inactivate RNase enzymes.

### Immunolabeling

The immunolabeling analysis was undertaken to examine the co-localization of native/endogenous PV and transgenic eGFP expression in DRGs from PVeGFP mice. The DRGs were removed as above (without dissociation) and emersion-fixed in 4% depolymerized paraformaldehyde (in phosphate buffer solution—PBS, pH 7.3) for 2–3 h. This tissue was subsequently treated with Dimethyl Sulfoxide (DMSO, Thermo Fisher Scientific) to permeabilize cellular membranes (3 × 15 mins) and then dehydrated in 100% ethanol (3 × 15 mins, Thermo Fisher Scientific). The tissue was submerged in poly-ethylene glycol (PEG-1000 molecular weight, Acros Organics) for 2 h, before setting in PEG 1450 and subsequent sectioning (20 μm) on a microtome. The sections were incubated in primary antibodies (polyclonal chicken anti-GFP 1:500, polyclonal rabbit anti-PV 1:400, Sapphire Bioscience Pty Ltd) diluted in a hypertonic PBS containing 10% donkey serum at room temperature for 15–18 h. After washing (3 × 15 mins in PBS), sections were incubated in species-specific secondary antibodies conjugated to Cy3 and FITC fluorophores (1:50, Jackson ImmunoResearch Pty Ltd) at room temperature for 2 h. The secondary antibody was removed by washing (3 × 15 min in PBS) and slices were mounted on glass slides using buffered glycerol (33% 0.5M Na_2_CO_3_ pH 8.6 in glycerol).

In addition to the above PV vs. GFP comparison, DRGs from wildtype (C57Bl/6) mice were processed to immunolabel and compare PV-expression with HCN 1, HCN 2, and HCN 4 expression. HCN 3 was not included in this analysis as antibodies to this subunit were found to only yield non-specific labeling. A total of three adult male C57Bl6 mice (20–22 g) were deeply anesthetized with pentobarbitone and perfused transcardially with 4% depolymerized formaldehyde. Lumbar DRG were removed and post-fixed in the same solution for an additional 2 h. Free-floating sections of ganglia (60 μm thick) were cut on a vibratome and subsequently incubated in 50% ethanol for 30 min to enhance antibody penetration. The sections were then incubated in a cocktail of primary antibodies containing guinea pig anti-PV (1:500; Frontier Institute Cat# PV-GP-Af1000, RRID:AB_2336938) with either rabbit anti HCN1 (1:250; Alomone Labs Cat# APC-056, RRID:AB_2039900), rabbit anti-HCN2 (1:500; Alomone Labs Cat# APC-030, RRID:AB_2313726) or mouse anti-HCN4 (diluted 1:500; UC Davis/NIH NeuroMab Facility Cat# 73–150, RRID:AB_10673158). Immunolabeling for PV was visualized using a goat anti-guinea pig secondary antibody conjugated to Alexa488, and the HCN subunits were visualized using the tyramide signal amplification approach described previously (Hughes et al., 2012). All primary and secondary antibody cocktails were made up of 0.3 M phosphate-buffered saline with 0.3% Triton X-100. Sections were incubated in primary antibodies for 72 h and in secondary antibodies for 12–18 h at 4°C.

Sections were cover-slipped and examined on an epifluorescence microscope (Olympus BX51) or confocal microscope (Zeiss LSM710, Hemel Hempstead, United Kingdom). Single images were captured using either a 10× or 20× objective in epifluorescence analysis. In confocal analysis, representative sections from each animal were scanned with image stacks collected using a 20× objective (0.9 digital zoom, 1 μm z-separation). Resulting images were analyzed off-line using Neurolucida for Confocal software (MicroBrightField, Colchester, VT, USA) to determine the expression of GFP, HCN1, 2 or 4 immunolabeling in PV-expressing cells.

### Electrophysiology Experiments

Recording micropipettes were made from borosilicate glass (1.5 mm OD, Harvard Apparatus, Kent, UK) and filled with a HEPES based internal solution containing in mM: 124 K-gluconate, 10 phosphocreatine di tris salt, 10 HEPES, 0.2 EGTA, 4 Mg2ATP, and 0.3 Na2GTP (pH 7.3, adjusted with KOH; Wang et al., 1994; Xu et al., 1997; Hayar et al., 2008). Series resistance (R_S_), membrane capacitance, and input resistance (R_IN_) were assessed based on the response to a hyperpolarizing 5 mV voltage step. These values were monitored throughout each experiment and when R_IN_ and/or R_S_ changed by more than 20% or >25 MΩ, respectively, cells were excluded from the analysis. Recordings were made at room temperature (22–24°C) and membrane potentials were not corrected for liquid junction potential.

To study APs, a depolarizing step protocol (100 pA increments, 800 ms duration) was applied to cells in the current clamp recording mode. Rheobase current was calculated as the smallest current step that evoked an AP. The AP generated for each cell at rheobase was used to measure AP threshold, rise time, amplitude, and width. AP threshold was determined on an expanded time scale by identifying the inflection point where membrane potential change exceeded 15 V/s. AP rise time was measured as 10–90% of the duration between AP threshold and the maximum positive peak, AP amplitude was the voltage difference between AP threshold and the maximum positive peak, and AP width was the duration of the positive spike at AP threshold. A derivative method was also used to differentiate between monophasic and biphasic repolarization phases on differentiated Rheobase APs. Previous work has shown biphasic repolarization reflects a depolarizing hump that is characteristic of nociceptive DRGs and can be used to identify this class. To test for the presence of I_h_, a current clamp protocol delivered several hyperpolarizing steps of increasing amplitude (−100 pA increments, 800 ms duration, −50 mV holding potential). If I_h_ was observed, a voltage clamp protocol was applied (10 mV steps, 800 ms duration, −50 mV holding potential) to the maximum amplitude of −110 mV. Current amplitude and activation kinetics were assessed using Axograph X analysis software.

#### Pharmacology

The identity of the hyperpolarization-evoked currents as I_h_ current was confirmed by the addition of 4-(N-Ethyl-N-phenylamino)-1,2 dimethyl-6-(methylamino) pyrimidinium chloride (ZD7288, Sigma-Aldrich), to the bath at a final concentration of 100 μM. In these experiments, a single −300 pA step (800 ms every 8.5 s) was applied to the cell in the current-clamp. This step was sufficient to monitor the I_h_ block during the ZD7288 application. Although some work indicates ZD7288 also affects Na channels (Wu et al., 2012), this off-target effect did not influence the large hyperpolarizing current step responses we assess.

### Molecular Experiments

#### Cell Collection

Using DEPC treated solutions, DRGs were removed and dissociated as described above. From the resulting suspension, 200–300 μl was plated on a plastic coverslip. Cells were allowed to settle for 15–20 min before the excess fluid was replaced with fresh HEPES buffer. GFP+ cells were visualized under fluorescent microscopy and collected, *via* gentle suction, into a glass micropipette (filled with DEPC HEPES) mounted on a micromanipulator. Once 50 cells were collected, they were expelled *via* positive pressure into a 2 ml Eppendorf tube. This process was repeated to collect 50 GFP− cells.

#### RNA Extraction and DNase Digestion

RNA extraction and purification from the 50-cell samples were completed as per the protocol “Cells” included in the RNeasy Micro Handbook (2nd Edition, Qiagen, 2007), using the solutions included in an RNeasy Micro kit (Qiagen Cat No 74004). RNA concentrations were then determined using a Nanodrop-1000 spectrophotometer (ThermoFisher Scientific Inc, Waltham, MA USA). This process provided approximately 10 μl of RNA sample to be used for reverse transcription (RT). To eliminate residual genomic DNA contamination, a DNA digestion step was performed at room temperature for 15 min after RNA extraction. In PCR grade microtubes (Scientific Specialties Incorporated, Lodi, CA, USA), 5 μl of the RNA samples were incubated with 1 μl DNase and 1 μl DNase 10× Buffer (both included in Invitrogen™ DNase I, Kit I) and 3 μl Nuclease free water (Qiagen). This process was halted by adding 1 μl of EDTA (25 mM, Invitrogen™ DNase I Kit I) and heating the samples in a thermocycler (Eppendorf, South Pacific) at 65°C for 10 min.

#### Reverse Transcription (RT)

For RT, a 5 μl aliquot from each of the DNA-free RNA samples (GFP+/−) was obtained for cDNA reactions. Oligo dT primers and Deoxynucleotide triphosphates (dNTPs, both from Bioline Pty Ltd.) as well as random hexamers (GeneWorks Pty Ltd, Adelaide, SA Australia) were added to the RT reaction and processed in a thermocycler for 5 min at 65°C before being cooled to 4°C and placed on ice for 1 min. Following this, 4 μl of 5× First Strand buffer, 1 μl RNase inhibitor, 1 μl Dithiothreitol (DTT, all from Bioline Pty Ltd.), as well as 1 μl reverse transcriptase (Superscript III, Invitrogen), was added before running in a thermocycler at 50°C for 60 mins, then 75 °C for 15 mins. For quality control, each RT sample was paired with a reverse transcriptase absent sample (called RT+ and RT−, respectively), where the 3 μl volume was replaced with Nuclease free water (Qiagen).

#### Qualitative Polymerase Chain Reaction (qPCR)

Based on RNA spectrophotometer readings, the cDNA samples were diluted in nuclease-free water to a final concentration of 0.2 ng/μl. Forward and reverse 5 prime (5′) primers for the four HCN subunits (Horwitz et al., 2011), GFP (Klein et al., 2000) and ß-actin were included (see Table 1). Separate qPCR master mixes were made for each gene to be investigated (GFP, ß-actin and HCN 1–4). Each master mix contained forward and reverse primers for the target gene at a final concentration of 20 μM. The SYBR green-based master mix from Bioline was used for all qPCRs. Each qPCR reaction comprised 7 μl of the master mix, primers, and water, plus 5 μl of cDNA sample for a total reaction volume of 12 μl. All reactions were run on an ABI 7500 Real-Time Thermal Cycler (ThermoFisher Scientific, Australia). After an initial polymerase activation step at 95°C for 10 min, there were 40 amplification cycles comprising a 95°C, 15 s denaturation step, followed by a 60°C 1 min annealing and extension step. All samples from a single animal (GFP+ and GFP−) were run in triplicate on the same qPCR plate. In addition to the RT+ and RT− samples, water controls were included on all plates, where nuclease-free water was substituted for cDNA.

**Table 1:** Primer sequence details.

Primer	Sequence	Supplier
HCN1	Forward—ACATGCTGTGCATTGGTTATGGCG Reverse—AACAAACATTGCGTAGCAGGTGGC	GeneWorks Pty Ltd
HCN2	Forward—ACTTCCGCACCGGCATTGTTATTG Reverse—TCGATTCCCTTCTTCACTATGAGG	GeneWorks Pty Ltd
HCN3	Forward—CCTCATCCGCTACATACACCAGT Reverse—GACACAGAGCAACATCATTCC	GeneWorks Pty Ltd
HCN4	Forward—GCATGATGCTTCTGCTGTGTCACT Reverse—TTCACCATGCCATTGATGGACACC	GeneWorks Pty Ltd
Beta Actin	Forward—ATGGAGGGGAATACAGCCC Reverse—TTCTTTGCAGCTCCTTCGTT	GeneWorks Pty Ltd
eGFP	Forward—ATCATGGCCGACAAGCAGAAGAAC Reverse—GTACAGCTCGTCCATGCCGAGAGT	GeneWorks Pty Ltd

### Analysis

For the electrophysiological data, statistical analysis was carried out using SPSS v10 (SPSS Inc., Chicago, IL, USA). Following normality checks (Shapiro–Wilks), independent *t*-tests and non-parametric Mann-Whitney tests were applied as appropriate across each measurement. The qPCR analysis was undertaken on paired samples of GFP+ and GFP− cells (*n* = 50). Each sample was tested in triplicate using forward and reverse primers for GFP and HCN 1–4 and the internal control gene β actin. Mean cycle threshold (Ct) values were determined for each triplicate (from each animal) and were used to determine ΔCt by subtracting the mean Ct of the internal control (β actin) from the mean Ct of the target primer (i.e., GFP and HCN 1–4). The resulting ΔCt values were analyzed for normality using a Shapiro–Wilks test. Normality was satisfied in all comparison groups except HCN 3. Thus, a two-tailed paired sample *T*-test was applied to the HCN 1, 2 and 4, while a nonparametric Wilcoxon test was applied to HCN 3 to compare GFP+ and GFP− groups. ΔCt values were converted to fold-change values between groups for each primer. Statistical significance was set at *p* < 0.05. All values are presented in the text as means ± SEM.

## Results

### Co-localization of Green Fluorescent Protein and Endogenous Parvalbumin

**Figure 1:**
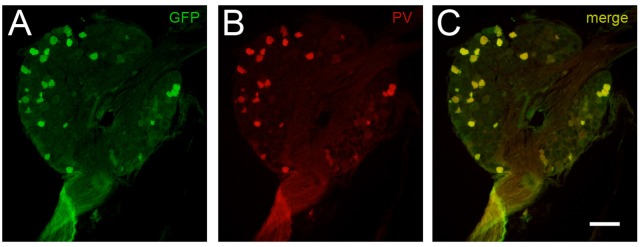
Comparison of GFP-expression and parvalbumin (PV) immunolabeling in dorsal root ganglia (DRG) cells. The fidelity of GFP expression in PV-expressing DRG neurons was tested to confirm the utility of tissue from the PVeGFP transgenic mouse line. Panels **(A,B)** show a representative DRG section immunolabeled for GFP (green) and PV (red), respectively. Overlaid image **(C)** shows co-localization (yellow) of GFP and endogenous PV in DRG cells. This analysis showed complete overlap (100%) in 645 DRG cells examined (*n* = 3 animals). Scale **(A–C)** = 100 μm.

To first confirm reliable and selective GFP expression, DRGs were extracted and immunolabeled for PV and GFP. Co-localization between PV immunoreactive neurons and GFP expression occurred in 100% of the 645 cells analyzed from PVGFP mice (*n* = 3, two DRGs per mouse). Additionally, we found that there were no instances of cells expressing GFP or PV in isolation (see Figure 1). These data show the PVeGFP transgenic mouse line reliably marks sensory neurons expressing PV.

### Electrophysiological Properties

**Figure 2:**
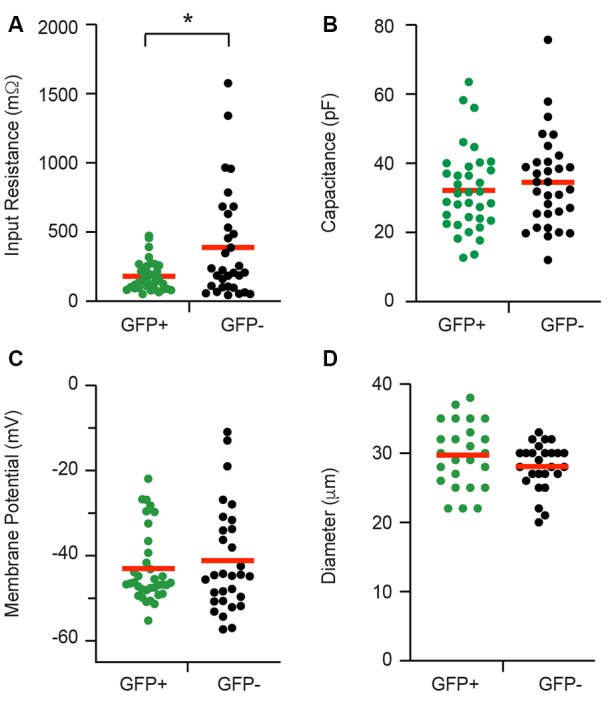
Passive electrical properties of proprioceptive DRG cells. Baseline properties of green fluorescent protein positive (GFP+) proprioceptive neurons and unidentified green fluorescent protein negative (GFP−) neurons were assessed during patch-clamp recordings. Scatter plots compare passive properties, including input resistance **(A)**, membrane capacitance **(B)**, resting membrane potential** (C)**, and cell diameter **(D)**. Values for GFP+ cells are shown in green while GFP− data appear black. Red horizontal bars indicate the mean for each group. Input resistance was the only property that differed between the two groups and was significantly lower in GFP+ recordings (*p* = 0.008). *p ≤ 0.05.

Whole cell patch clamp recordings were made from isolated cells taken from 21 adult PV-eGFP mice (average yield = 3.1 recordings per animal). The data (presented herein as GFP+ vs. GFP−) for passive membrane properties are summarized in Figure 2. Input resistance was lower in GFP+ cells when compared with GFP− cells (152.18 ± 18.34 vs. 388.63 ± 68.79 MΩ, *p* = 0.01, *n* = 35 and 32, respectively). Series resistance was similar in GFP+ and GFP− cells (12.0 ± 0.5 vs. 12.4 ± 0.6 MΩ, *p* = 0.64, *n* = 35 and 32, respectively, data not shown). Membrane capacitance (32.16 ± 2.02 vs. 34.47 ± 2.34 pF, *p* = 0.46, *n* = 35 and 32, respectively) and resting membrane potential (−42.99 ± 1.43 and −41.14 ± 2.26 mV, *p* = 0.88, *n* = 35 and 32, respectively) were similar in GFP+ and GFP− cells. Cell diameter (29.72 ± 0.94 vs. 28.08 ± 0.67 μm, *p* = 0.21, *n* = 25 and 26, respectively), as measured *via* a calibrated scale bar, was also similar in both populations.

**Figure 3:**
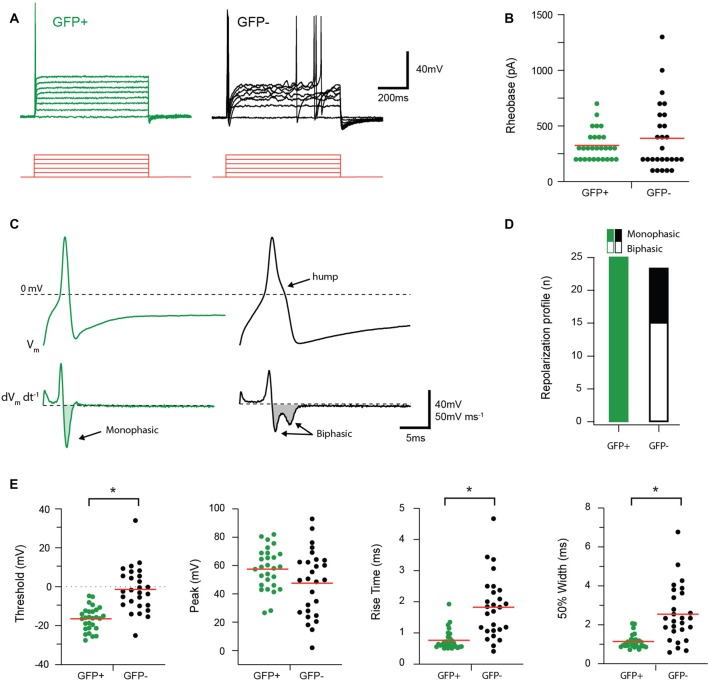
Discharge and action potential (AP) properties of proprioceptive DRG cells. Two types of AP discharge were observed in DRG neurons during step current injections (100 pA increments, 800 ms duration, red traces). **(A)** Overlaid traces show a typical response from GFP+ and GFP− cells, contrasting a single spike response at the onset of current injection in GFP+ cells with multi-spiking responses observed in some GFP− recordings (7/24). **(B)** Scatter plot shows group data comparing Rheobase in GFP+ (green circles) and GFP− (black circles) cells. While group means (red lines) are similar for both groups, the spread, and range of data in GFP− cells is greater. **(C)** Upper traces show rheobase APs from a GFP+ (green trace) and GFP− cell (black trace) on an expanded time scale. Note while APs exhibit a similar amplitude, the time courses are distinct, with GFP+ cells exhibiting faster APs and a distinct hump appearing on the repolarization phase in the GFP− trace. Lower traces are differentiated AP data (from above) clearly identifying the distinct repolarization phases of GFP+ and GFP− cells as monophasic and biphasic waveforms, respectively. **(D)** Bar graph summarizes relative incidence of cells exhibiting monophasic (filled bar) and biphasic (open bar) differentiated waveforms. All GFP+ cells exhibited monophasic waveforms, whereas more than half the GFP− cells exhibited biphasic responses (15/24). **(E)** Scatter plots show group data comparing AP properties in GFP+ (green circles) and GFP− (black circles) cells. AP height was similar in GFP+ and GFP− cells, but AP threshold, rise time, and half-width differed in the two cell types, with faster kinetics and more hyperpolarized thresholds in the GFP+ sample (*p* < 0.05). *p ≤ 0.05.

AP discharge was assessed in response to multiple depolarizing step injections in the recorded cell as shown in Figure 3. The resulting AP discharge was classified as either single (SS) or multiple spiking (MS). All GFP+ cells exhibited the SS discharge profile (25/25 cells), whereas only 70% (17/24 cells) of the GFP− population exhibited the SS spiking profile. The remaining GFP− cells fired multiple spikes that were a mix of phasic bursting and tonic firing phenotypes. The properties of the first AP generated in response to current injection (rheobase) were also compared between groups. No differences in rheobase current (325 ± 25.52 vs. 388.89 ± 52.79 pA, *p* = 0.80, *n* = 28 and 27, respectively) or AP peak amplitude (57.55 ± 2.76 vs. 47.64 ± 4.53 mV, *p* = 0.06, *n* = 28 and 27, respectively) were identified between GFP+ and GFP− cells. In contrast, AP threshold occurred at more hyperpolarized potentials (−16.44 ± 1.15 vs. −1.40 ± 2.23 mV *p* = 0.01, *n* = 28 and 27, respectively); rise time was faster (0.77 ± 0.06 vs. 1.83 ± 0.19 ms, *p* = 0.00, *n* = 28 and 27, respectively); and AP half-width was significantly shorter (1.15 ± 0.07 vs. 2.55 ± 0.28 ms, *p* = 0.00, *n* = 28 and 27, respectively) in GFP+ recordings compared with the GFP− population. Rheobase AP traces were also differentiated to assess the presence of biphasic repolarization, which has been shown to reflect a depolarizing hump featured in APs from nociceptive afferents (Figure 3C). This assessment showed that all GFP+ cells exhibited a monophasic repolarization, whereas 15/24 GFP− cells exhibited biphasic responses (Figure 3D).

**Figure 4:**
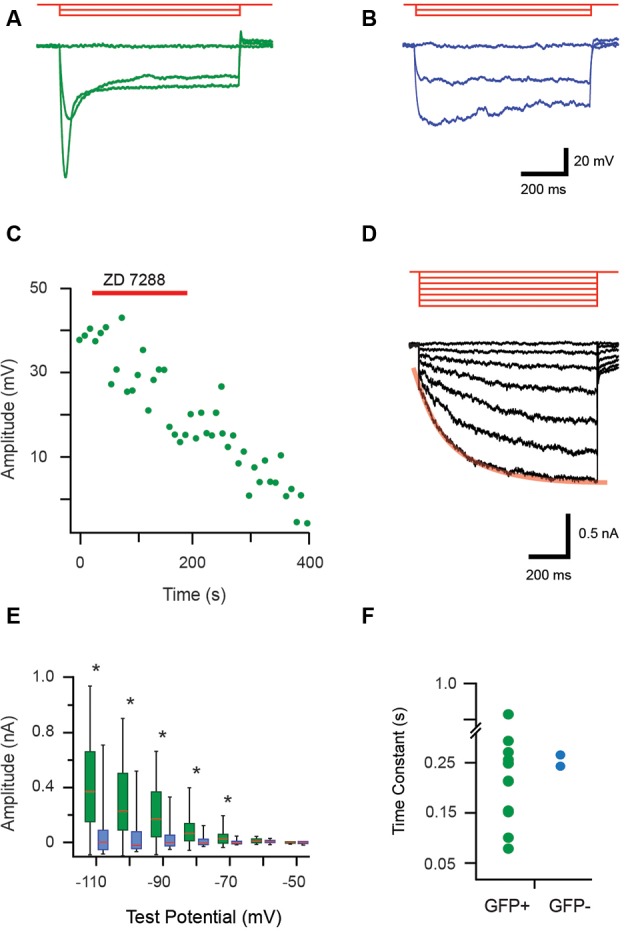
Properties of hyperpolarization-activated currents (I_h_) in proprioceptive DRG cells. **(A,B)** Overlaid traces showing current-clamp recordings from isolated GFP+ and GFP− DRG neurons during hyperpolarizing current injection (3 steps, 100 pA increments, 1 s duration, red traces). All GFP+ cells exhibited a prominent sag characterized by a rapid return towards resting membrane potential that became more prominent as hyperpolarized step injection amplitude increased **(A)**. This profile is associated with a hyperpolarization activated current, termed I_h_. In contrast, few GFP− cells exhibited evidence of the I_h_ current and when present it was minimal (**B**, sag less prominent). **(C)** Bath application of an antagonist (ZD7288, 100 μM) was used to confirm the identity of I_h_ in current-clamp mode. Peak I_h_ voltage amplitude, measured every 8.5 s in response to a 300 pA hyperpolarizing current step was sensitive to the addition of ZD7288 (100 μM), which abolished I_h_ responses. **(D)** Overlaid traces show I_h_ currents recorded from a GFP+ cell during a voltage clamp protocol (−10 mV steps, holding potential −50 mV, red traces). These responses feature a prominent voltage-activated current that becomes more pronounced as hyperpolarization is increased. Note red line shows an exponential fit to the I_h_ activation profile during the largest hyperpolarizing step. **(E)** Box and whisker plot shows group data comparing I_h_ amplitude in GFP+ (green) and GFP− (blue) cells from the hyperpolarizing step responses (shown in **D**). I_h_ amplitude is minimal until steps reach −70 mV before hyperpolarization I_h_ amplitude begins to increase substantially in GFP+ neurons (*p* < 0.005). **(F)** An exponential was fit to the largest I_h_ current trace (i.e., −110 mV step, overlaid red line) providing a time constant (or, activation rate) for I_h_ in GFP+ (green) and the two GFP− cells (blue). Activation rates varied markedly for GFP+ cells while the two GFP− cells had similar values that fell within the range of GFP+ activation rates. *p ≤ 0.05.

During current-clamp recordings hyperpolarizing current step injection responses often featured a “sag” in membrane potential, which then returned towards RMP, consistent with an I_h_ current (Figures 4A,B). All recordings that assessed this feature confirmed GFP+ cells exhibited I_h_-like currents (18/18 recordings), whereas these currents were only detected in 11% (2/17) of the GFP− cells. The identity of this current was verified in a subset of recordings by testing ZD7288 (100 μM) sensitivity, a commonly used blocker of I_h_ (Figure 4C, *n* = 3). I_h_ currents were also studying in voltage-clamp using a protocol that stepped membrane potential from −50 mV to more hyperpolarized potentials in 10 mV increments (Figure 4D). The average I_h_ amplitude for each voltage step was calculated as the difference between values immediately after the onset and conclusion of seven hyperpolarizing steps (−50 to −110 mV). The box-whisker plots (Figure 4E) show average I_h_ amplitude did not differ for the first two voltage steps (to −50 and −60 mV) in GFP+ and GFP− cells (*p* = 0.70 and 0.16, respectively). However, I_h_ currents were much larger in GFP+ cells for the steps between −70 and −110 mV. This is indicative of the presence of substantial I_h_ in GFP+ cells (−70 mV *p* = 0.001, −80 mV *p* = 0.002, −90 mV *p* = 0.004, −100 mV *p* = 0.003 and −110 mV *p* = 0.003).

The activation rate of I_h_ was also assessed by fitting an exponential over 10%–90% of the maximum current amplitude (i.e., the −110 mV step) for GFP+ and GFP− cells (Figure 4D). The resulting I_h_ activation time constant values varied markedly for GFP+ cells (0.08–0.83 s). Values for the two GFP− cells that expressed I_h_ had similar activation rates (0.27 and 0.24 s) and fell within the range expressed by the GFP+ cells.

### Gene Expression: qPCR Ct Analysis for HCN Channel Subunits

**Figure 5:**
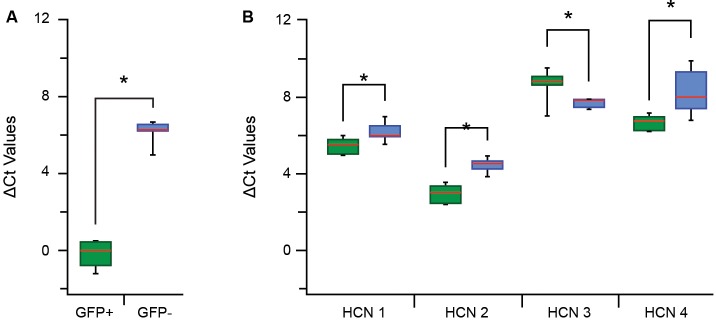
Quantitative polymerase chain reaction (qPCR) ΔCt analysis for hyperpolarization-activated cyclic nucleotide-gated channel (HCN channel) subunits in proprioceptive DRG cells. **(A)** Box and whisker plot show group data comparing ΔCt for GFP expression in GFP+ (green) and GFP− (blue) samples (normalized to β actin expression). The low GFP ΔCt in GFP+, but not GFP−, confirm high expression in this sample and the reliability of this sampling approach. **(B)** Box whisker plots plot shows group data comparing ΔCt values for HCN1–4 in GFP+ (green) and GFP− (blue) samples. The expression of all HCN subunits was significantly different between GFP+ and GFP− groups (where *p < 0.05). HCN 2 had the greatest overall expression in both samples whilst HCN 3 and 4 were both expressed at much lower levels. Expression levels were highest for HCN 2, then HCN 1 and HCN 4 in the GFP+ sample and these values all exceeded corresponding values in the GFP− samples. In contrast, HCN 3 expression was lower in the GFP+ sample vs. GFP− cells.

We next examined the mRNA expression levels of HCN 1–4 subunits to gain insight into the molecular determinants of the I_h_ currents and activation rates reported above (Figure 4F). A qPCR analysis was first undertaken on paired samples of pooled GFP+ vs. GFP− cells for specific GFP primers to confirm our isolation procedures faithfully captured populations of GFP+ and GFP− cells (Figure 5A). Mean Ct values show there was a large difference in ΔCt values for the GFP+ and GFP− sample (−0.11 ± 0.25 vs. 6.19 ± 0.15, *p* > 0.01). When expressed as a fold difference, there was >90 times more GFP expression in our GFP+ vs. GFP− sample. This confirmed our procedures provided a highly enriched sample of PV positive (GFP-expressing putative proprioceptive) cells for subsequent analysis of HCN subunit expression.

Regarding HCN subunit mRNA expression, significantly higher levels of HCN 1, 2 and 4 were detected in the GFP+ vs. GFP− sample, reflected in lower ΔCt values (Figure 5B) and equating to 1,5-, 3-, and 3.5-fold differences, respectively. In contrast, HCN 3 was the only subunit to show lower expression in the GFP+ vs. GFP− sample (ΔCt = 8.7 ± 0.3 vs. 7.7 ± 0.1, *p* = 0.03; Figure 5B), representing a 0.5-fold difference. Within each group, HCN 2 was the most highly expressed subunit in GFP+ and GFP− samples. For GFP+ cells, HCN 2 expression was highest, followed by HCN 1 then HCN 4 (ΔCt = 2.96 ± 0.16 vs. 5.49 ± 0.150 vs. 7.35 ± 0.15).

### HCN Channel Subunit Expression in PV DRG Neurons

**Figure 6:**
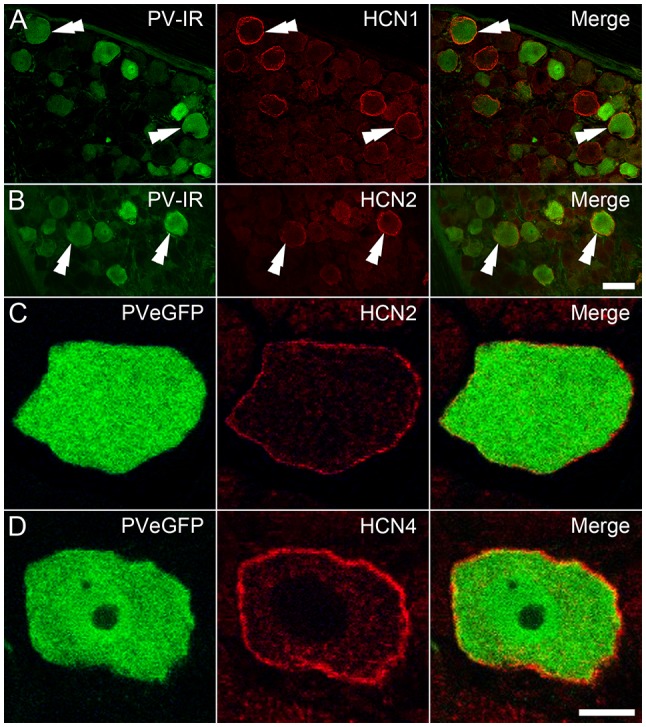
Immunohistochemical localization of HCN subunits in PV-expressing DRG neurons. **(A,B)** PV-IR DRG neurons (green) in wild-type mice were shown to express both HCN1 (**A**; red) and HCN2 (**B**; red), with examples denoted by double arrowheads. **(C,D)** In DRGs from PVeGFP mice, GFP-expressing cells (green) showed immunolabeling for both HCN2 (**C**; red) and HCN4 (**D**; red). Immunolabeling for each of the HCN subunits was restricted to the cell membrane. Scale Bars (μm): **(A,B)** = 50; **(C,D)** = 10.

To assess the impact of the above findings at a protein level, DRGs from wildtype tissue were immunolabeled for PV and HCN subunit expression. PV positive DRG cells were identified by immunolabeling in the cytoplasm of subpopulations of DRG neurons, whereas that for HCN1 and HCN2 was confined to membranes of cell bodies and axons in restricted subsets of cells (Figures 6A,B). In contrast, immunolabeling for HCN4 was expressed in the cytoplasm, and therefore deemed nonspecific. In earlier pilot experiments using tissue from PVeGFP mice, HCN2 and HCN4 immunolabeling were confined to membranes and axons of GFP labeled DRG neurons, but not analyzed (Figures 6C,D). Nevertheless, this observation confirms some PV positive DRG neurons also express HCN4 in their membranes, albeit without formal quantification. Regarding HCN1 and HCN2 subunit labeling that could be analyzed, less than half of PV-IR neurons expressed immunolabeling for HCN1 (mean 43.8% ± 8.8; 15/34 PV cells; *n* = 2 animals), whereas HCN2 was expressed in half than half the PV-IR sample (mean 65.3% ± 11.0; 48/75 PV cells; *n* = 3). These observations confirm that the HCN subunit mRNA identified in qPCR experiments does translate to functional protein in PV DRG cells, and the relative incidence of HCN1 and HCN2 subunit expression among PV cells also mirrors the mRNA levels detected in this population.

## Discussion

This study used targeted patch-clamp electrophysiology and molecular analysis of HCN channel subtypes to compare the properties of putative proprioceptive neurons (GFP+) with a group of non-proprioceptive “other” sensory populations (GFP−). Our main electrophysiology findings are that GFP+ and GFP− neurons differed in their active and I_h_ properties. Molecular analysis, supported by immunolabeling, showed expression of HCN 1, 2 and 4 subunits were higher in GFP+ neurons. In contrast, HCN 3 subunits, though expressed at low levels, were more highly expressed in GFP− cells. Below, we discuss the caveats associated with our experiments and our main findings in terms of their relevance to proprioceptive signaling.

During depolarizing current step injection, GFP+ neurons always exhibited a single spike phenotype whereas GFP− neurons showed a mix of single and MS (Figure 3). The phasic firing and strong adaptation in GFP+ neurons are consistent with the *in vivo* responses of sensory neurons that innervate Pacinian corpuscles and muscle spindles (Lawson, 2005). Both receptor types are classed as low threshold mechanoreceptors, have fast conduction velocities, and are best suited to signaling rapid changes in pressure and muscle length, respectively. Such signaling would require AP discharge at the onset or offset of a stimulus. Their phasic discharge (Figure 3A), however, is at odds with the known ability of muscle spindle afferents to support high-frequency firing, especially at the onset and cessation of muscle lengthening.

This above discrepancy presumably relates to a number of factors. First, the use of a long square step stimulus does not reflect the way neurons are excited *in vivo* with different stimulus profiles shown to produce different responses depending on other neuronal populations (Graham et al., 2004). Second, our recordings were made from the soma of isolated sensory neurons vs. an intact preparation. *In vivo*, AP generation occurs in peripheral terminals and is conducted in a centrally-projecting axon that is linked to the soma of sensory neurons. The excitability of the terminal and axon of proprioceptive fibers, missing in our preparation, maybe more important for determining the firing pattern. Nevertheless, reduced somatic DRG preparations continue to be a useful approach to assay the proteins and channels expressed by different afferent types. Finally, there is evidence that neurons can undergo changes in reduced or isolated preparations that potentially alter the suite of ion channels that support AP generation and/or repetitive discharge (Turrigiano et al., 1994; Hayar et al., 2008; Werner et al., 2012). Future experiments that deliver short depolarizing steps at high frequency, ramps, and stimuli with other profiles would allow exploration of the capacity of GFP+ neurons to support firing modes and frequencies observed *in vivo*. In the future, it may also be possible to record directly from the peripheral terminals of GFP− labeled proprioceptors. These would be heroic experiments, however, such recordings have been made from the terminals of peripheral sensory nerves in the cornea (Carr et al., 2009).

In addition to the above differences in patterns of discharge, there were also marked differences in individual AP characteristics between the two groups of neurons in our study. APs in GFP+ neurons had faster kinetics, based on AP rise time and half-width (Figure 3C). This finding is consistent with data from numerous studies showing large-diameter neurons (like the proprioceptive population) generate narrow APs and respond to much lower stimulation intensities than other DRG cell types (Fang et al., 2005). These properties are also consistent with a role in proprioceptive signaling, where fast and repetitive AP generation is required in response to changes in muscle length (muscle spindles) and/or force (Golgi tendon organs). Similarly, the broader APs in GFP− neurons match the function of high threshold receptors, such as nociceptors, recorded from intact DRG neurons *in vivo* (Djouhri et al., 1998).

Our assessment of I_h_ showed this current to be present in all GFP+ neurons vs. only ~10% of GFP− neurons. This is consistent with early work which showed significant I_h_ in most large sensory neurons (putative proprioceptors; Scroggs et al., 1994). It also fits with the role hyperpolarization-activated cationic currents play in high-frequency AP discharge, as occurs in the spindle and GTO afferents (Moosmang et al., 2001; Stevens et al., 2001; Notomi and Shigemoto, 2004; Baruscotti et al., 2005). As multiple HCN channel subunits exist and shape the properties of I_h_ currents, we investigated both amplitude and activation rates of this current (based on fitting exponentials to current onset; Figure 4D). GFP+ neurons exhibited a range of I_h_ amplitudes (Figure 4E) and activation rates (Figure 4F), consistent with the expression of different HCN channels/subunits (Acosta et al., 2012; Hughes et al., 2013). Molecular analysis showed all four HCN subunits could be detected in isolated sensory neurons (both GFP+ and GFP− neurons; Figure 5). However, expression profiles were distinct between the GFP+ and GFP− samples. Specifically, HCN 2, 1, and 4 expressions (listed in descending levels based on ΔCt values; Figure 5B) were higher in GFP+ vs. GFP− neurons. Alternatively, HCN 3 expression was higher in the GFP− population. Recent data on HCN 3 subunits (Lainez et al., 2019) suggest the expression of HCN 3 in the GFP− population is not important for (small) c-fiber afferents, rather it plays a role in setting excitability in medium-sized neurons that conduct in the δ and Aβ range (i.e., not muscle spindle and GTO afferents; Lawson, 2002). This finding marries well with the similar cell diameters of GFP and GFP− neurons in our sample. It also suggests or recordings in GFP− neurons may have been biased towards the larger δ and Aβ DRG cell types, as opposed to the much smaller c-fiber cell population. In contrast, HCN 2 and 1 were highly expressed in GFP+ neurons These observations were also validated at the protein level, with immunolabeling for HCN1, 2, and 4 resolved in PV positive DRG cells. Furthermore, the proportion of PV DRG cells expressing HCN 1 and 2 mirrored mRNA levels and suggested a degree of heterogeneity in subunit expression among proprioceptive afferents identified by PV expression.

Expression patterns favoring HCN1 and 2 subunits also agree with functional data indicating these subunits exhibit the fastest activation rates of the four HCN isoforms (Jiang et al., 2008), correlating with the fast I_h_ activation times we recorded in many GFP+ neurons (Figure 4F). This also fits with the high *in vivo* discharge rates recorded in proprioceptors because fast HCN channel activation rates elevate resting potential and AP firing frequency (Pape and McCormick, 1989; Pape, 1996; Ludwig et al., 2003; Nolan et al., 2003; Chan et al., 2004; Momin et al., 2008). The higher levels of HCN 4 in GFP+ neurons (vs. GFP− neurons) also match a proposed association between these subunits with high discharge rates (Hughes et al., 2013).

Of course, our examination of the contribution of I_h_ and HCN subunits to GFP+ and GFP− neuron function comes with several caveats. First, we could not determine if the peripheral axon of GFP+ neurons was in fact connected to a proprioceptor (muscle spindle or GTO) due to the dissociated nature of the preparation employed here. This limitation could be addressed in future experiments by making recordings from an *ex vivo* preparation consisting of a DRG—peripheral nerve—peripheral organ. Such preparations were originally developed to study sensory neurons connected to tactile and nociceptive afferents in the skin (Woodbury et al., 2001). More recently an attached *ex vivo* muscle preparation was used to study nociceptors in the muscle (Jankowski et al., 2013). The use of an *ex vivo* muscle preparation in combination with stimuli that selectively activate muscle spindles or GTOs (e.g., ramped muscle stretch) and targeted recording from neurons in DRGs from the PVGFP mouse could address this issue. Achieving visualized high-resolution patch-clamp recordings from GFP+ neurons would also necessitate gentle treatment of DRGs with enzymes that loosen connective tissue and allow for patch pipette access as successfully employed in other ganglia (Yawo, 1989).

A second limitation relates to the population contrasted against the GFP+ proprioceptive neurons, which was a mixed population selected at random, presumably including recordings from a range of afferent types. This would have limited our ability to resolve distinct proprioceptive properties from a mixed control sample. Testing the responsiveness of control (GFP−) recordings to modality-specific agonists such as capsaicin, icilin, menthol, chloroquine, or temperature changes could be used to further dissect this population. Despite this, our analysis of the falling phase of APs did detect biphasic repolarization in many GFP− cells, confirming a depolarization hump associated with nociceptive afferents. Given the similarity of soma size between samples, these are more likely to represent δ than c-fiber nociceptors. Finally, our examination of the HCN subunit expression was undertaken on populations of pooled cells. Thus, the higher expression could reflect elevated expression across one sample or very high expression in a subset of cells. Our immunolabeling data goes some way to addressing this issue, showing that only subsets of PV cells express HCN1 and 2 (~45% vs. 65%, respectively), supporting heterogeneity within the PV population. This could be further explored by characterizing I_h_ properties in *single* dissociated neurons (GFP+ and GFP−) and subsequent single-cell qPCR analysis for HCN expression. These data would also speak to our conclusion that a range of I_h_ amplitudes and activation rates (Figure 4) implies distinct proprioceptive types can be differentiated within GFP+ neurons, based on their HCN channel profiles.

In summary, this study was motivated by the notion that distinct properties in a sensory neuron population are likely to impart modality-specific function and as such, may represent targets to alter function in that neuron population. This strategy’s value has been highlighted in the successful targeting of sensory neurons involved in nociception and pain (Haberberger et al., 2019). We asked what properties might distinguish proprioceptive neurons from other afferents because a better understanding of these features will be relevant to the well-known age-related decline in proprioceptive function and the increased incidence of falls (Greaves et al., 1991; Kim et al., 2007; Rosant et al., 2007; Wingert et al., 2014). Our analysis of proprioceptive neurons, identified by PV expression, show they indeed have a range of different properties to putative non-proprioceptive neurons. However, the proprioceptive neuron population was not homogeneous, at least based on the expression of HCN channel subtypes. Notwithstanding these limitations, our study provides a foundation for future studies on the excitability of proprioceptive afferents and how they change with age and/or under sensorimotor pathologies.

## Data Availability Statement

All datasets generated for this study are included in the article.

## Ethics Statement

All experimental procedures performed at the University of Newcastle were approved by The University of Newcastle Animal Care and Ethics Committee under Section 25 of the NSW Animal Research Act, 1985. All experimental procedures performed at the University of Glasgow were conducted in accordance with the European Community directive 86/609/EEC and UK Animals (Scientific Procedures) Act 1986.

## Author Contributions

JM, RC, PJ, DH, DS, and BG conceived and designed the research study. JM, OD, and KB conducted experiments and acquired data. JM, TB, JI, RC, OD, KB, and BG analyzed data. JM, TB, RC, DH, and BG wrote the manuscript. All authors edited the final version of the manuscript.

## Conflict of Interest

The authors declare that the research was conducted in the absence of any commercial or financial relationships that could be construed as a potential conflict of interest.

## Abbreviations

AP: Action Potential
Ct: Cycle Threshold
DH: Dorsal Horn
dNTP: Deoxynucleotide triphosphates
DRG: Dorsal Root Ganglia
DTT: Dithiothreitol
EGTA: ethylene glycol-bis(β-aminoethyl ether)-N,N,N′,N′-tetraacetic acid
GFP+: Green Fluorescent Protein Positive
GFP−: Green Fluorescent Protein Negative
HCN channel: hyperpolarization-activated cyclic nucleotide-gated channel
I_h_: Hyperpolarization-Activated Current
PBS: Phosphate Buffer Solution
PEG: Poly-Ethylene Glycol
PV: Parvalbumin
qPCR: Quantitative Polymerase Chain Reaction
RT: Reverse Transcription
RT+: Reverse transcription with reverse transcriptase
RT−: Reverse transcription without reverse transcriptase
sACSF: Substituted Artificial Cerebrospinal Fluid
SEM: Standard Error of the Mean
TrkC: Tyrosine Receptor Kinase C
ZD7288: 4-(N-Ethyl-N-phenylamino)-1,2 dimethyl-6-(methylamino) pyrimidinium chloride.
